# An Integrated Multi-Omics Study Revealed Metabolic Alterations Underlying the Effects of Coffee Consumption

**DOI:** 10.1371/journal.pone.0091134

**Published:** 2014-03-11

**Authors:** Shoko Takahashi, Kenji Saito, Huijuan Jia, Hisanori Kato

**Affiliations:** 1 Department of Applied Biological Chemistry, Graduate School of Agricultural and Life Sciences, the University of Tokyo, Tokyo, Japan; 2 Food for Life, Organization for Interdisciplinary Research Projects, the University of Tokyo, Tokyo, Japan; Hosptial Infantil Universitario Niño Jesús, CIBEROBN, Spain

## Abstract

Many epidemiological studies have indicated that coffee consumption may reduce the risks of developing obesity and diabetes, but the underlying mechanisms of these effects are poorly understood. Our previous study revealed the changes on gene expression profiles in the livers of C57BL/6J mice fed a high-fat diet containing three types of coffee (caffeinated, decaffeinated and green unroasted coffee), using DNA microarrays. The results revealed remarkable alterations in lipid metabolism-related molecules which may be involved in the anti-obesity effects of coffee. We conducted the present study to further elucidate the metabolic alterations underlying the effects of coffee consumption through comprehensive proteomic and metabolomic analyses. Proteomics revealed an up-regulation of isocitrate dehydrogenase (a key enzyme in the TCA cycle) and its related proteins, suggesting increased energy generation. The metabolomics showed an up-regulation of metabolites involved in the urea cycle, with which the transcriptome data were highly consistent, indicating accelerated energy expenditure. The TCA cycle and the urea cycle are likely be accelerated in a concerted manner, since they are directly connected by mutually providing each other's intermediates. The up-regulation of these pathways might result in a metabolic shift causing increased ATP turnover, which is related to the alterations of lipid metabolism. This mechanism may play an important part in the suppressive effects of coffee consumption on obesity, inflammation, and hepatosteatosis. This study newly revealed global metabolic alterations induced by coffee intake, providing significant insights into the association between coffee intake and the prevention of type 2 diabetes, utilizing the benefits of multi-omics analyses.

## Introduction

Lines of evidence have shown that the chronic consumption of coffee may reduce the risk of some diseases such as obesity and diabetes [1], [2]. However, despite the abundance of epidemiological studies indicating such beneficial effects [3], [4], the information on the underlying mechanisms is limited. Considering the fact that coffee is now one of the most popular beverages in the world, biomolecular studies of the health benefits of coffee should be of great significance to the maintenance and promotion of human health. The goal of the present study was to reveal in an exhaustive manner the fundamental metabolic alterations caused by coffee consumption.

More strategic and more systematic approaches to studies of the functionality of food and food components are needed, since the mechanisms underlying their effects are poorly understood in many foods, including coffee. Since food components affect the status of the whole body by influencing arrays of transcripts, proteins and metabolites, new research areas have been developed based on the studies of such groups of molecules. These research areas are called by the name of object or field studied, suffixed by “omics,” such as transcriptomics, proteomics and metabolomics [5], [6]. These various ‘omics’ technologies enable researchers in the field of food and nutrition to comprehensively understand the response of the body to diets, to discover novel functions of food factors, and to elucidate unknown mechanisms of the effects of nutrients. These technologies also proved themselves to be effective for investigating safety issues related to foods [7]. Transcriptomic analyses using DNA microarrays are widely used due to their efficiency and comprehensiveness in omics research [8].

Although many studies have addressed the impact of food components at the transcriptomic level, their findings should be interpreted with reservation since they provide information only on changes in mRNA abundance. Proteomic analyses reveal the changes at the protein level, which are the direct players in cellular regulation and homeostasis. In addition to transcriptomics and proteomics, metabolomics, the use of which is still relatively limited in the field of food science, is of importance for the comprehensive understanding of the influence of food factors. Metabolomics using CE-TOF MS aims at determining as many metabolites present in organisms as possible [9].

These omics studies enable us to understand physiological information at the respective levels of mRNA, protein, and metabolite. Considering the highly distinct and diverse features of information obtained from each omics platform, one could expect that combinations of different omics should provide highly comprehensive views on the effects of, for instance, nutrition and diets. Such an attempt of combining different omics is referred to as multiple omics or “multi-omics,” integrated omics. Since the number of examples of integrated omics research is limited — especially in the field of food science — the first nutritional omics study of the effects of coffee by a combination of three omics analyses is of high significance. The study will also be valuable as a precedent for future studies of the functionality of foods whose mechanisms are as yet unknown.

There is a need for comparisons of laboratory findings with the epidemiological data concerning the anti-obesity and anti-diabetes effects of coffee. In our previous study examining the effects of three types of coffee (caffeinated, decaffeinated, and green unroasted coffee) on the livers of C57BL/6J mice fed a high-fat diet, we obtained transcriptome data using a DNA microarray [2]. The three types of coffee suppressed the overweight and fat accumulation induced by a high-fat diet throughout the experimental period, without affecting calorie intake. The transcriptomics results suggested the alterations in lipid metabolism-related molecules as one of the factors mediating the anti-obesity effect of coffee, which may lead to the prevention of type 2 diabetes.

We conducted the present study to delve further into the effects of coffee, and the results revealed the alterations at the levels of proteome and metabolome by coffee. We further interpreted the results through the integration with the previous transcriptome data to more extensively clarify the metabolic alterations underlying the anti-obesity and anti-diabetic effects of coffee consumption via a multi-omics study.

## Results

Body weights, organ weights, biochemical tests and the DNA microarray were previously reported [2]. Briefly, the results showed that the three types of coffee suppressed the weight gain and fat accumulation induced by a high-fat diet, and the amount of triacylglycerol in the liver of all coffee groups showed significant decreases. Furthermore, decreased hepatic expression of the genes for proteins that play pivotal roles in hepatic steatosis were revealed by DNA microarray. All microarray data have been submitted to Gene Expression Omnibus (accession number GSE53131).

### TCA cycle-related proteins were up-regulated in the coffee groups

**Figure 1:**
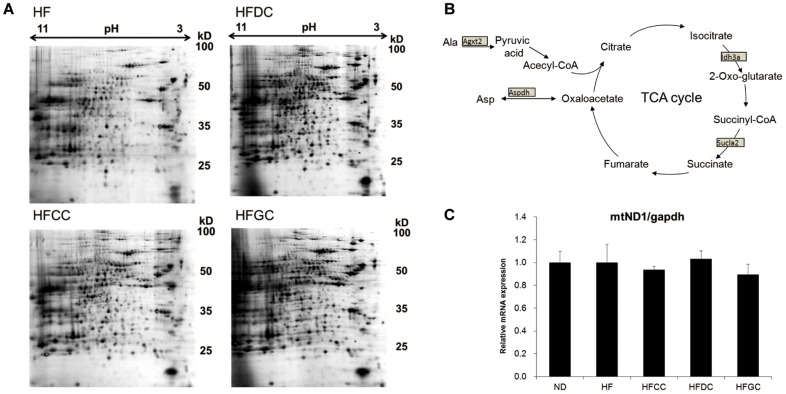
Proteomics revealing the up-regulation of TCA cycle-related proteins in the coffee groups. (**A**) Representative 2DE images obtained from livers of each groups. 2DE was performed using dry strips with a pH range of 3–11 for the first dimension and SDS-PAGE for the second dimension using 450 µg of protein extract. The gels were then stained using Flamingo gel staining dye. Calibration of molecular weight and pI was performed using PDQuest software. (**B**) Changes in TCA cycle-related proteins are shown in the boxes.

To broadly identify proteins whose levels were significantly affected by the intake of coffee, we performed two-dimensional electrophoresis (2DE) differential analyses of mouse livers of the HF group and coffee groups. We detected the proteome maps in 2DE gels by Flamingo gel staining dye (Fig. 1A). The detected spots were quantitatively analyzed with PDQuest software, and statistical comparisons were made by Student's t-test (*p*<0.05, n = 3).

Among the differentially expressed spots in 2DE, the numbers of identified proteins whose expression was affected by the intake of each type of coffee are shown in Table S2. Successfully identified proteins in the HFCC (high-fat diet with caffeinated coffee), HFDC (high-fat diet with decaffeinated coffee) and HFGC (high-fat diet with green unroasted coffee) groups as well as the HF (high-fat diet) group are listed in Tables 1–3. In the HFCC group, alterations in several proteins related to glucose metabolism were observed compared to the HF group, including the up-regulation of glycine N-methyltransferase, isoform CRA_a (GNMT) and the down-regulations of fructose-1,6-bisphosphatase 1 (FBP1) and regucalcin (Table 1). The consumption of DC (decaffeinated coffee) influenced many TCA cycle-related proteins, including alanine-glyoxylate aminotransferase 2, isoform CRA_b, L-aspartate dehydrogenase, and isocitrate dehydrogenase 3 alpha, isoform CRA_d, which is a rate-limiting enzyme of the TCA cycle (Table 2). The protein alteration in one of the rate-limiting enzymes in TCA cycle was found in HFDC group, but the similar tendency of increment in isocitrate dehydrogenase 3 alpha was observed in HFCC and HFGC group.

**Table 1:** List of identified proteins differentially expressed in livers of the HFCC group.

NCBI gi	Identified protein	Exp. pI^1^	Exp. Mr^2^	Theo. Mr ^3^	HFCC vs. HF
gi | 148703895	esterase D/formylglutathione hydrolase, isoform CRA_a	7.8	29.8	30261	Up
gi | 148691587	glycine N-methyltransferase, isoform CRA_a	7.8	31.1	29077	Up
gi | 6754212	heme oxygenase 1	7.5	24.3	32965	Up
gi | 21312002	putative L-aspartate dehydrogenase	7.6	27.9	30479	Up
gi | 123258683	pericentrin 1	6.8	34	32514	Up
gi | 9506589	fructose-1,6-bisphosphatase 1	6.8	35.2	37311	Down
gi | 309265190	alpha-enolase-like isoform 11	6.6	46.4	47640	Up
gi | 6677739	regucalcin	4.5	31	33899	Down
gi | 50510617	eukaryotic translation initiation factor 5B	9.2	29.5	37847	Up

HFCC: high-fat caffeinated coffee diet; HF: high-fat diet. Up: The protein abundance was increased by coffee compared to the HF group, Down: The protein abundance was decreased by coffee compared to the HF group. ^1^Experimental value of pI. ^2^Experimental value of molecular mass (kDa). ^3^Theoretical value of molecular mass (Da).

**Table 2:** List of identified proteins differentially expressed in livers of HFDC group.

NCBI gi	Identified protein	Exp. pI^1^	Exp. Mr^2^	Theo. Mr^3^	HFDC vs. HF
gi | 148703895	esterase D/formylglutathione hydrolase, isoform CRA_a	7.8	29.8	30261	Up
gi | 6754212	heme oxygenase 1	7.5	24.3	32965	Up
gi | 21312002	putative L-aspartate dehydrogenase	7.6	27.9	30479	Up
gi | 50510617	eukaryotic translation initiation factor 5B	9.2	29.5	37847	Down
gi | 148671356	alanine-glyoxylate aminotransferase 2, isoform CRA_b	8.1	21.7	23538	Up
gi | 309265176	alpha-enolase-like isoform 7	7.3	47.1	47931	Down
gi | 148708069	NADH dehydrogenase (ubiquinone) 1 alpha subcomplex 10, isoform CRA_f	6.9	39.6	40863	Up
gi | 8393866	ornithine aminotransferase, mitochondrial precursor	6.2	44.4	48723	Up
gi | 200952	Selenium-binding liver protein	6.2	52.9	52889	Down
gi | 20071222	NADH dehydrogenase (ubiquinone) Fe-S protein 3	5.7	25.1	30358	Up
gi | 6679299	prohibitin	5.7	25.9	29859	Up
gi | 148705362	ketohexokinase, isoform CRA_c	5.6	28.8	27997	Up
gi | 148693874	isocitrate dehydrogenase 3 alpha, isoform CRA_d	5.6	37.1	39752	Up

See the Table 1 legend. HFDC: high-fat decaffeinated coffee diet.

**Table 3:** List of identified proteins differentially expressed in livers of HFGC group.

NCBI gi	Identified protein	Exp. pI^1^	Exp. Mr^2^	Theo. Mr^3^	HFGC vs HF
gi | 148703895	esterase D/formylglutathione hydrolase, isoform CRA_a	7.8	29.8	30261	Up
gi | 21312002	putative L-aspartate dehydrogenase	7.6	27.9	30479	Up
gi | 123258683	pericentrin 1	6.8	34	32514	Up
gi | 50510617	eukaryotic translation initiation factor 5B	9.2	29.5	37847	Down
gi | 148671356	alanine-glyoxylate aminotransferase 2, isoform CRA_b	8.1	21.7	23538	Up
gi | 309265176	alpha-enolase-like isoform 7	7.3	47.1	47931	Down
gi | 148708069	NADH dehydrogenase (ubiquinone) 1 alpha subcomplex 10, isoform CRA_f	6.9	39.6	40863	Up
gi | 6679299	prohibitin	5.7	25.9	29859	Up
gi | 148705362	ketohexokinase, isoform CRA_c	5.6	28.8	27997	Up
gi | 3766201	ATP-specific succinyl-CoA synthetase beta subunit	5.5	42.2	46557	Up
gi | 26330031	unnamed protein product	7.3	40.7	44613	Up

See the Table 1 legend. HFGC: high-fat green unroasted caffeinated coffee diet.

Proteins related to the electron transport system, NADH dehydrogenase (ubiquinone) 1 alpha subcomplex 10, isoform CRA_f and NADH dehydrogenase (ubiquinone) Fe-S protein 3 were also up-regulated in the HFDC group compared to the HF group (Table 2). The consumption of GC (green unroasted coffee) caused the same changes in L-aspartate dehydrogenase and alanine-glyoxylate aminotransferase 2 as DC did, and it resulted in the up-regulation of a subunit of another TCA cycle enzyme, ATP-specific succinyl-CoA synthetase beta subunit (Table 3). The relationship among these changes in TCA cycle-related proteins is shown in Figure 1B.

Since mitochondrial proteins including TCA cycle-related proteins were up-regulated in the livers of the coffee-consuming mice, we measured the expression levels of mitochondrial DNA by quantitative real-time PCR, which give an indication of the number of mitochondria. The results showed no significant differences among the diet groups (Fig. 1C).

### Metabolome analysis revealed alterations in the urea cycle

We performed a metabolome analysis to explore the hepatic metabolic alterations underlying the effects of coffee consumption. Among the peaks obtained from the CE-TOF MS analysis, 287 peaks were identified according to the value of m/z and MT from metabolite database. Of these metabolites, 165 peaks were detected by cationic mode and 122 peaks were detected by anionic mode. The numbers of identified metabolites whose expression was affected by the intake of different types of coffee are shown in Table S3.

**Figure 2:**
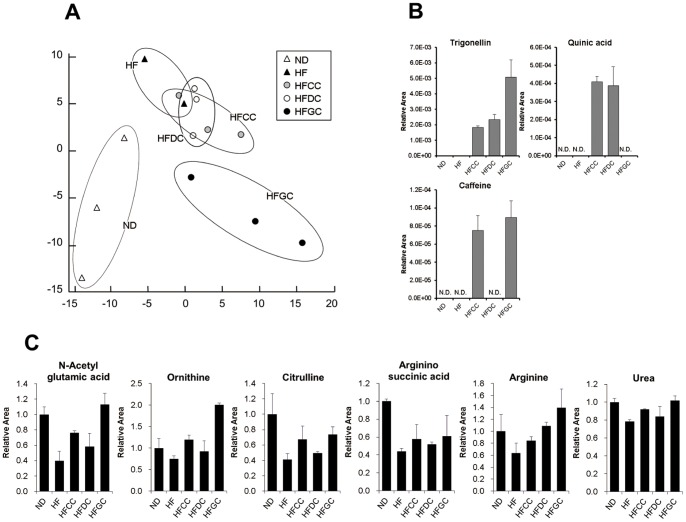
Metabolome analysis revealing the alterations in the urea cycle. (**A**) PCA (principal component analysis) of metabolomics datasets of the livers in each group. The PCA analysis was performed with the detected peaks by using SampleStat ver.3.14. (**B**) Relative peak areas of coffee-derived metabolites. Mean values with their standard errors of the relative peak areas of these metabolites are shown. (**C**) Relative peak areas of urea cycle-related metabolites. Mean values with their standard errors of the relative peak areas of these metabolites are shown.

A PCA (principal component analysis) was performed using all peaks in order to grasp the rough picture of the impact of each diet on the metabolome (Fig. 2A). The results revealed that principle component 1 tended to distinguish the ND (normal diet) group and the high-fat diet groups, including the HF group and coffee groups. Principle component 2 tended to separate the HF group and coffee groups. The coffee groups — especially the green coffee group — were separated from the HF group.

Among the identified metabolites, caffeine, trigonelline and quinic acid derive from coffee powder. The relative peak areas of these metabolites are shown in Figure 2B. Caffeine was detected only in the livers of the mice in the HFCC and HFGC groups. Trigonelline was detected in all coffee groups, among which the HFGC group had a higher content than the other coffee groups. Quinic acid was detected only in the HFCC and HFDC groups.

Since the principle component 2 separated the HF group and the coffee groups in the PCA, we focused on the metabolites related to the second component, and we found that several metabolites related to the urea cycle were consistently affected by HF and coffee. The relative peak areas of these metabolites are shown in Figure 2C. N-acetylglutamate (N-AcGlu), which is a positive regulator of the urea cycle, was decreased by the high-fat diet but increased by GC mice compared to the HF group. Similar changes were found in the abundance of ornithine, citruline, argininosuccinate (ArgSuccinate), and arginine, which are the intermediates of the urea cycle, and the final product, urea.

### Integrated analysis revealed the overview of metabolic status induced by coffee intake

**Figure 3:**
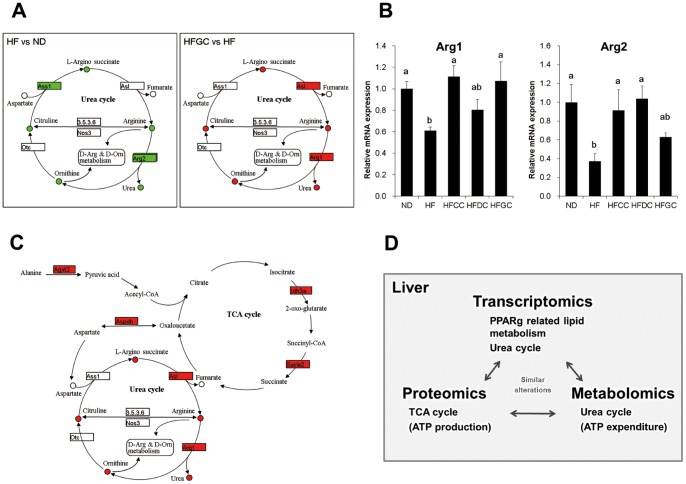
Integrated analysis of transcriptomics, metabolomics and proteomics. (**A**) Transcriptome and metabolome data were mapped onto KEGG (Kyoto Encyclopedia of Genes and Genomes, http://www.genome.jp/kegg/) pathways on the web. (**B**) Effects of coffee on the mRNA expression of urea cycle genes analyzed by qPCR. The total RNA samples obtained from mice fed a high-fat diet with or without coffee were subjected to qPCR. Mean values with their standard errors of relative mRNA expression are shown. Mean values with asterisks are significantly different compared to the HF group by the Tukey-Kramer test (*p<0.05, **p<0.01). (**C**) “Whole picture” of the metabolic alterations in the TCA cycle and the urea cycle. The relationship among these changes in the TCA cycle- and urea cycle-relevant molecules is shown. (**D**) Summary of the findings from the present study.

When previous transcriptomic data and metabolome data were uploaded together and visualized in the KEGG (Kyoto Encyclopedia of Genes and Genomes, http://www.genome.jp/kegg/) pathway, the alterations within and around the urea cycle were found to be highly consistent between transcripts and metabolites (Fig. 3A). From these results, it is apparent that the expressions of the genes related to the urea cycle were down-regulated by the high-fat diet and up-regulated by coffee consumption, although we had not paid much attention to these changes when highly represented gene groups were sorted out by using an interpretative phenomenological analysis (IPA). The data shown in Figure 3A are the representative data of the HFGC group, and the HFCC and HFDC mice showed similar alterations (data not shown). The mRNA expression changes were validated by quantitative real-time PCR (Fig. 3B), which was consistent with the microarray data.

The overview of metabolic changes caused by coffee consumption is shown in Figure 3C, based on the relationship among urea cycle-related alterations shown by metabolomics and transcriptomics, and the TCA cycle-related alterations obtained by proteomics.

## Discussion

Nowadays coffee is of rising interest to scientists as a food with broad-ranging functionalities, and it is well known that the chronic intake of coffee could reduce the risk of obesity and diabetes [10]. Recently it has been reported that pure chlorogenic acids and pure caffeine are the compounds which have the anti-obesity and anti-diabetic effect [18], [19], although there are a large variety compounds in coffee probably in bioactive concentrations. The result of the present study suggests that caffeine is neither necessary nor sufficient to induce lower body weight, decrease adipose depots, and decrease hepatic lipids, since decaffeinated coffee with low caffeine concentrations also had great effect similarly to caffeinated coffee. On the other hand, approximately 0.42% of chlorogenic acids in HFGC group had similar effects to the previously reported study, in which 0.5% of pure chlorogenic acids had anti-obesity effect on C57BL/6J mice. It was suggested that there are certain contribution of chlorogenic acids to the effect of coffee.

The present study was conducted to reveal the fundamental metabolic alterations related to the beneficial effects of coffee through comprehensive analytical technologies. Hepatic fat accumulation, which is one of the important upstream events of type 2 diabetes, was suppressed by the intake of coffee in C57BL/6J mice fed a high-fat diet [2]. In order to also explore the changes in adipose tissue, the mRNA expression levels of some genes relating to lipid metabolism, including *FAS*, *SCD1*, *ACC1*, *ACC2*, *SREBP1c*, were measured using total RNA extracted from the white adipose tissue sample. Inconsistent with the results in previously reported studies [1], [18], there were no alterations in the mRNA expression levels of these genes in adipose tissue (data not shown). It is well known that hepatic TG accumulation induced by high-fat diet causes VLDL release from liver, and fatty acids are taken up by adipose tissue via the action of lipoprotein lipase. Although remarkable mRNA expression changes in adipose tissue were not observed in the previous study, it is considered that the remarkable metabolic alterations in the livers must have certain effects on adipose tissue. In the present study, the liver tissue was first analyzed considering the fact that liver functions and hepatic lipid composition are strongly affected by food factors, and also liver steatosis and adipose tissue disorder are deeply related.

In proteomics using two-dimensional electrophoresis combined with MALDI-TOF mass spectrometry, we focused on proteins that exhibited significant changes, although the numbers of identified proteins were limited in the present proteome analysis (Tables 1–3). Our results indicate that the expressions of many identified proteins involved in the TCA cycle were increased by the intake of coffee. Thus, it can be considered that the production of ATP via the TCA cycle was enhanced by the intake of coffee. L-aspartate dehydrogenase, which was increased in all coffee groups compared to the HF group (Tables 1–3), is an enzyme that catalyzes the conversion of aspartate to oxaloacetate. The deamination of aspartate results in oxaloacetate, an intermediate of the TCA cycle (Fig. 1B). Alanine-glyoxylate aminotransferase 2, isoform CRA_b, increased in the HFDC and HFGC groups, is a transferase that catalyzes alanine and glyoxylate to produce pyruvate and glycine. The protein abundance of isocitrate dehydrogenase 3 alpha is known as one of the rate-limiting enzymes of the TCA cycle. NADH dehydrogenase (ubiquinone) 1 alpha subcomplex 10, isoform CRA_f and NADH dehydrogenase (ubiquinone) Fe-S protein 3 (Table 2) are the proteins known to catalyze the reaction of electron transport from NADH to ubiquinone. The up-regulation of these proteins suggested that the consumption of NADH produced in the TCA cycle were increased through the intake of coffee. These results suggested that coffee consumption enhanced energy production. The rate of TCA cycle turnover, which is related to important energy metabolism, has been reported to be reduced in the liver of mice fed high-fat diet [15], and the present study revealed the preventive effect of coffee on it, which might play a pivotal role in the effect of coffee intake on energy metabolism.

However, although many proteins related to mitochondrial proteins were up-regulated, there were no significant differences in the number of mitochondria (Fig. 1C). It therefore appears that the intake of coffee had an influence on the alterations in mitochondrial proteins without affecting the number of mitochondria. On the other hand, no significant alterations in the mRNA levels of TCA cycle-related genes (data not shown) were found in the transcriptome data. These observations exemplify the importance of combining analyses at the levels of proteins and transcripts.

From the results of principle component analysis in the metabolomics through CE-TOF MS (Fig. 2A), we observed that the high-fat diet had a significant influence on the metabolite profiles of the mouse livers, since the ND group and HF group were clearly distinguishable. The intake of coffee also had a strong influence on the hepatic metabolite profiles, especially the green unroasted coffee. The relative peak areas of the coffee-derived metabolites in Figure 2B showed the reliability of this metabolome analysis, since the results were consistent with those expected from the difference of ingredients.

Urea production is reported to be decreased due to dysfunction of liver enzymes in diet-induced obese animals [11], and urea cycle-related metabolites were significantly affected by the consumption of the high-fat diet and coffee (Fig. 2C). These alterations in metabolites include arginine, ornithine, citruline, and urea. N-AcGlu, a positive regulator of the urea cycle, also showed similar changes. The similar tendency of increment in N-acetylglutamate and other metabolites in urea cycle were observed (Fig. 2C). Since HFGC group showed the largest increases in their abundance, these changes is likely to be attributable to coffee polyphenol. These data, highly consistent with the transcriptomic data (Fig. 3A), are suggesting that the depressed urea cycle in the HF diet groups was enhanced by coffee consumption. An acceleration of energy expenditure is thought to have occurred in all of the coffee groups.

The TCA cycle and the urea cycle are directly connected as they mutually provide the other's intermediates [12]. Therefore, the up-regulation of both the TCA cycle and the urea cycle in this study resulted in increased ATP turnover, which may be related to the alterations of lipid metabolism (Fig. 3C). The mechanism may play an important part in the suppressive effects of coffee consumption on obesity, and eventually diabetes. These global metabolic alterations in the TCA cycle and the urea cycle induced by coffee intake provided significant insights into the association of coffee intake and enhanced energy consumption, and they may contribute to a reduction in the risk of obesity which may ultimately lead to the prevention of type 2 diabetes. It also demonstrated the benefits of combined omics approaches in food and nutrition science.

Although the alterations in lipid metabolism related molecules were revealed in the experiment of DNA microarray, the changes were not observed in protein level or metabolite level. One of the reasons for this discrepancy is much lower comprehensiveness of proteomics and metabolomics techniques as compared with transcriptomics using DNA microarray. Therefore the number of proteins identified to be differentially changed was not so large compared with that of trancripts. However, the clues from each analysis complemented each other, successfully leading to the crucial findings of the present study Furthermore, the results from different omics analyses do not necessarily consistent partly because the timelines for the alterations of mRNA, protein, and metabolites are different. The present study showed the potency of the combination of different omics even with limited comprehensiveness. In addition to the insights obtained by transcriptomic analysis, other patterns of regulations have emerged in our study, some of which were unique to proteome data — the TCA cycle, and another common to all — the urea cycle (Fig. 3D). Our integrated approach on the effects of coffee intake using multiple omics techniques is a pioneering study, especially in regard to the effect of food factors. The complete elucidation of the mechanisms of functional foods may prove to be challenging, since the effects of food factors are relatively mild. As such, the integration of multiple layers of information provided by the multi-omics approach will continue to gain importance.

## Materials and Methods

### Animal experiments

We conducted an animal experiment to evaluate the effects of the intake of coffee, as described [2]. Briefly, 7-week-old male C57BL/6J mice purchased from Charles River Laboratories Japan (Yokohama) were divided into the following five groups (n = 8−9). The normal diet group (ND group) was fed D12450B (10 kcal% fat, Research Diets, New Brunswick, NJ, USA). The high-fat diet group (HF group) was fed D12492 (60 kcal% fat, Research Diets, New Brunswick, NJ, USA). The caffeinated coffee group (HFCC group) was fed a high-fat diet containing 2% caffeinated freeze-dried coffee. The decaffeinated coffee group (HFDC group) was fed a high-fat diet containing 2% decaffeinated freeze-dried coffee. The green unroasted coffee group (HFGC group) was fed a high-fat diet containing 2% unroasted caffeinated freeze-dried coffee. The 2% coffee powder is equivalent to approximately 4 cups (2 g coffee powder/140 mL/cup) of coffee per day in humans, which was calculated using a formula for dose translation based on body surface area [16]. This dose was employed since epidemiologic data have noted that 3–4 cups of coffee suppresses the risk of type 2 diabetes [17]. The content of caffeine in caffeinated, decaffeinated, and green unroasted coffee powder are 2.5, 0.1 and 2.5 g/100 g, and the content of total polyphenols are 15, 14, and 21 g/100 g, respectively [2]. The mice had ad libitum access to their diets and drinking water. After 9 weeks, mice were sacrificed and the livers were then excised. All animal experiments were performed in accordance with the guidelines of the Animal Usage Committee of the Faculty of Agriculture of the University of Tokyo, and were verified by the committee (Approval number, P09-374).

### DNA microarray data

The DNA microarray data used in the present study were obtained in our previous study, which was carried out with the Affymetrix GeneChip Mouse Genome 430 2.0 array (Affymetrix, Santa Clara, CA, USA) [2].

### Proteome analysis

We performed the differential proteomic analysis of the mouse livers using two-dimensional electrophoresis (2DE) combined with matrix-assisted laser desorption/ionization-time of flight (MALDI-TOF) mass spectrometry in the same manner as in our previous study [13]. Briefly, 2DE was performed using dry strips with a pH range of 3–11 for the first dimension and sodium dodecyl sulfate-polyacrylamide gel electrophoresis (SDS-PAGE) for the second dimension using 450 µg of protein extract. The gels were then stained using Flamingo gel staining dye.

The calibration of molecular weight and pI was performed using the PDQuest 2-D analysis software package (Version 8.0.1, Bio-Rad, Hercules, CA). The spots showing significant changes in expression level were manually excised from the 2-DE gels and identified by peptide mass fingerprinting method. Using the MASCOT search program (http://www.matrixscience.com), we matched peptide masses with theoretical peptides of all proteins in the National Center for Biotechnology Information (NCBI) database.

### Metabolome analysis

Frozen mice liver samples (n = 2 in the HF group, n = 3 in the other groups) were transferred into 500 µL of methanol containing 50 µM of external standard. After homogenization by BMS-M10N21 (bms, Tokyo) at 1,500 rpm, 120 s five times, 500 µL of chloroform and 200 µL of ultra-pure water were added to the homogenate and mixed well and centrifuged at 2,300 g for 5 min at 4°C. The resultant water phases were ultrafiltrated by the Millipore Ultrafree-MC PLHCC HMT Centrifugal Filter Device, 5 kDa (Millipore, Billerica, MA). The filtrates were dried and dissolved in 50 µL of ultra-pure water.

We then subjected the samples obtained to capillary electrophoresis time-of-flight mass spectrometry (CE-TOFMS) analysis using the Agilent CE-TOFMS system (Agilent Technologies, Santa Clara, CA) at 4°C. The alignment of detected peaks was performed according to the m/z value and normalized migration time. We performed a principal component analysis (PCA) with the detected peaks using the statistical analysis software SampleStat ver.3.14 (Human Metabolome Technologies Inc., Tsuruoka, Japan), and we performed a hierarchical clustering analysis (HCA) using PeakStat ver.3.18 (Human Metabolome Technologies). The relative area value of each peak was calculated and used for the intergroup comparison. Samples that were obviously characterizing outliers were eliminated from the analysis.

### Quantitative real-time RT-PCR analysis

Total RNA was used for am mRNA analysis by quantitative real-time PCR. Primers were designed using a web application (PRIMER3), and their sequences are shown in Table S1. SYBR Green EX (Takara Bio, Madison, WI) was used on a real-time PCR detection system (Takara Bio). The relative amounts of mRNA were normalized to glyceraldehyde-3-phosphate dehydrogenase (GAPDH) and are expressed as the fold-change value.

### Integrated analysis of transcriptomics and metabolomics

We used the web-based tool Keggle (http://keggle.jp), a novel tool for the visualization of omics-data created by the author's group [14]. Transcriptome and metabolome data were mapped onto KEGG (Kyoto Encyclopedia of Genes and Genomes, http://www.genome.jp/kegg/) pathways on the web.

### Statistical tests

Data are presented as the means with standard errors for the metabolomics analysis, and the standard error of the mean for the RT-PCR results. Three randomly selected mice from each group were used for the three omics experiments. Statistical significance in the quantitative RT-PCR analysis was assessed using a one-way ANOVA followed by the Tukey-Kramer test for multiple comparisons. Significance was accepted at *p*<0.05.

## Supporting Information

Table S1 **PCR primer sequences of interest genes for detecting levels of mRNA expression.**
(DOCX)

Table S2 **The number of identified proteins whose expression was affected in the HFCC, HFDC and HFGC groups compared to the HF group.**
(DOCX)

Table S3 **The number of identified metabolites whose expression was affected in the HFCC, HFDC and HFGC groups compared to the HF group.**
(DOCX)

Table S4 **Diet information: Trigonelline, caffeine and polyphenol profile of coffee powder.** CC, Caffeinated coffee; DC, decaffeinated coffee; GC, green unroasted coffee.
(DOCX)
