# Supplementary material for: An Integrated Multi-Omics Study Revealed Metabolic Alterations Underlying the Effects of Coffee Consumption
Source: PLoS One. 2014 Mar 11;9(3):e91134. doi: 10.1371/journal.pone.0091134 (PMC3949743; doi:10.1371/journal.pone.0091134)
Supplement: Table S1 — PCR primer sequences of interest genes for detecting levels of mRNA expression. (DOCX) [file pone.0091134.s001.docx]

**Supplemental information**

**Table S1.** PCR primer sequences of interest genes for detecting levels of mRNA expression

|  | Forward | Reverse |
| --- | --- | --- |
| *ND1* | ATTACTTCTGCCAGCCTGAC | GGGTCCTAGGAAGATAATAGTTG |
| *Arg1* | AAGAATGGAAGAGTCAGTGTGGTG | TGGTTGTCAGGGGAGTGTTG |
| *Arg2* | TCTCCTCCACGGGCAAA | GCTGGACCATATTCCACTCCT |
| *Ass1* | CAGCCCAGATGTCCTTGAGATAG | TGTCAATGCGACCCACTCC |
| *Asl* | GCCATCCGGACCAGAAAA | TCGACAGCACCCACAAACC |
