# Supplementary material for: An Integrated Multi-Omics Study Revealed Metabolic Alterations Underlying the Effects of Coffee Consumption
Source: PLoS One. 2014 Mar 11;9(3):e91134. doi: 10.1371/journal.pone.0091134 (PMC3949743; doi:10.1371/journal.pone.0091134)
Supplement: Table S2 — The number of identified proteins whose expression was affected in the HFCC, HFDC and HFGC groups compared to the HF group. (DOCX) [file pone.0091134.s002.docx]

**Supplemental information**

**Table S2.** The number of identified proteins whose expression was affected in the HFCC, HFDC and HFGC groups compared to the HF group

|  | HFCC | HFDC | HFGC |
| --- | --- | --- | --- |
| Up | 7 | 10 | 9 |
| Down | 2 | 3 | 2 |
| Total | 9 | 13 | 11 |
