# Supplementary material for: An Integrated Multi-Omics Study Revealed Metabolic Alterations Underlying the Effects of Coffee Consumption
Source: PLoS One. 2014 Mar 11;9(3):e91134. doi: 10.1371/journal.pone.0091134 (PMC3949743; doi:10.1371/journal.pone.0091134)
Supplement: Table S4 — Diet information: Trigonelline, caffeine and polyphenol profile of coffee powder. CC, Caffeinated coffee; DC, decaffeinated coffee; GC, green unroasted coffee. (DOCX) [file pone.0091134.s004.docx]

**Supplemental information**

**Table S4.** Diet information: Trigonelline, caffeine and polyphenol profile of coffee powder.

|  |  |  |  | CC | DC | GC |
| --- | --- | --- | --- | --- | --- | --- |
| Trigonelline | |  |  | 0.78 | 0.88 | 2.00 |
| Caffeine | |  |  | 2.5 | 0.1 | 2.5 |
| Polyphenol | |  |  | 15 | 14 | 21 |
| Total chlorogenic acid | |  |  | 3.18 | 3.67 | 20.9 |
|  | 3-caffeoylquinic acid |  |  | 0.72 | 0.88 | 4.96 |
|  | 4-caffeoylquinic acid+3-feruloylquinic acid | | | 0.91 | 0.98 | 5.54 |
|  | 5-feruloylquinic acid |  |  | 0.96 | 1.28 | 5.65 |
|  | 4-feruloylquinic acid |  |  | 0.15 | 0.17 | 1.04 |
|  | 5-feruloylquinic acid |  |  | 0.29 | 0.23 | 1.32 |
|  | 3,4-dicaffeoylquinic acid |  |  | 0.07 | 0.07 | 0.94 |
|  | 3,5-dicaffeoylquinic acid |  |  | 0.03 | 0.02 | 0.56 |
|  | 4,5-dicaffeoylquinic acid |  |  | 0.05 | 0.05 | 0.99 |
|  |  |  |  |  |  | (g/100g) |

CC, Caffeinated coffee; DC, decaffeinated coffee; GC, green unroasted coffee.
